# S21. EVENT-RELATED REPETITIVE TMS TO RIGHT POSTERIOR STS (BUT NOT OCCIPITAL FACE AREA) IN HEALTHY VOLUNTEERS (HV) BRIEFLY RECAPITULATES FACE EMOTION RECOGNITION (FER) DEFICITS OF SCHIZOPHRENIA (SZ)

**DOI:** 10.1093/schbul/sby018.808

**Published:** 2018-04-01

**Authors:** Cheryl Corcoran, Jack Grinband, Jaimie Gowatsky, Casimir Klim, Matthew Hoptman, Daniel Javitt

**Affiliations:** 1 Icahn School of Medicine at Mount Sinai; 2 Columbia University, NYSPI; 3 NYSPI; 4 Nathan Kline Institute; 5 Columbia University, Nathan Kline Institute

## Abstract

**Background:**

Profound FER deficits exist in Sz, causing social disability, though can be partly remediated with computer-based training. Neurostimulation might augment remediation if critical nodes were identified. We aimed to 1) briefly recapitulate FER deficits of Sz in HV using rTMS to rpSTS, 2) identify connectivity patterns of rpSTS regressed by FER, and 3) apply TMS to rpSTS with fMRI as readout.

**Methods:**

1) Nine healthy volunteers had rTMS (10 Hz; 500 msec; 110% RMT) to rpSTS or rOFA (counterbalanced; 10/10 system overlay with standard MRI) concurrent (1/3 trials) with stimuli (http://faces.mpdl.mpg.de/) for emotion or gender identification (button press). 14 Sz patients completed these tasks without TMS. 2) Whole-brain resting-connectivity analyses, seeded by rpSTS, was applied in 27 Sz and 35 HV who also completed the UPenn FER task. 3) BOLD fMRI was obtained in 4 HV pre- and post-TMS to rpSTS (1 Hz; 20 minutes).

**Results:**

1) In HV, rTMS to rpSTS only (not OFA) significantly slowed reaction time for FER only (not gender identification): overall F test for logRT (p=.001) with post-hoc rpSTS vs.OFA (p=.005) and rpSTS vs. non-stim trials (p=.004). rpSTS recapitulated slowed RT ad lower FER accuracy of Sz. 2) In both HV and Sz, rpSTS had significant resting connectivity with V1 (p= .00013), positively modulated by FER accuracy. 3) Analyses are ongoing.

**Discussion:**

rpSTS is a critical node in the FER circuit with connectivity to primary visual cortex modulated by FER, whose disruption recapitulates FER deficits, making it a candidate target for remediatory neurostimulation.

